# The trend of phylogenetic and epitope variations of SARS-CoV-2 Omicron sub-lineages in Iran

**DOI:** 10.3389/fmicb.2024.1531712

**Published:** 2025-01-29

**Authors:** Mehdi Shabani, Ahmad Nejati, Jila Yavarian, Kaveh Sadeghi, Sevrin Zadheidar, Akram Sadat Ahmadi, Monire Ghadirali, Arghavan Zebardast, Adel Abedi, Mohammad Hossein Najmi, Nazanin-Zahra Shafiei-Jandaghi, Talat Mokhtari-Azad

**Affiliations:** ^1^Department of Virology, School of Public Health, Tehran University of Medical Sciences, Tehran, Iran; ^2^Research Center for Antibiotic Stewardship and Antimicrobial Resistance, Tehran University of Medical Sciences, Tehran, Iran; ^3^Department of Mathematics, Shahid Beheshti University, Tehran, Iran; ^4^Department of Bioinformatics, Faculty of Biological Sciences, Tarbiat Modares University, Tehran, Iran

**Keywords:** SARS-CoV-2, Omicron sub-lineages, phylogenetic, epitope variations, immunity

## Abstract

**Introduction:**

Severe acute respiratory syndrome coronavirus 2 (SARS-CoV-2) has been a significant public health issue worldwide in recent years. The most recently circulating variant of SARS-CoV-2, Omicron, and its rapid evolution into various sub-lineages have raised concerns regarding the effects of the immunity on the virus epitopes, in the human population. The present study evaluated and compared these important variations among different Omicron sub-lineages in Iran.

**Methodology:**

From October 2023 to August 2024, high coverage whole genome sequences of 49 SARS-CoV-2 strains were subjected to phylogenetic analysis and evaluation of B cell, CD4^+^, and CD8^+^ T cell epitopes in Iran National Influenza Centre.

**Results:**

The phylogenetic tree exhibited eight Nextstrain clades (21L, 22F, 23B, 23H, 23D, 24A, 24B, and 24C) in 48 studied strains, and one recombinant strain (XDK.1). The evaluation of B cell, CD4^+^, and CD8^+^ T cell epitopes in all studied strains revealed 31, 65, and 78%, of conservation, respectively. The low B cell epitopes conservation rate among Omicron sub-lineages underscored the escaping from neutralizing humoral immunity. T cell epitopes of the SARS-CoV-2 were considerably preserved across major Omicron sub-lineages. Conservation levels varied based on the epitope class (higher for CD8^+^ vs. CD4^+^), protein (higher for non-spike vs. spike), and clades (higher for 21L, 22F, 23B, 23H, 23D, and 24B vs. 24A and 24C).

**Conclusion:**

Herein, the increased conservation of CD8^+^ epitopes compared to CD4^+^ and B cell epitopes is probably attributable to the shorter length of the peptides associated with CD8^+^ epitopes. The high rate of T-cell epitopes conservation in non-spike proteins among different sub-lineages of the Omicron in this study highlighted the importance of cell-mediated immunity and suggested that non-spike proteins might be more attractive targets for future SARS-CoV-2 vaccines.

## Introduction

Severe acute respiratory syndrome coronavirus 2 (SARS-CoV-2), a highly transmissible and pathogenic coronavirus, emerged in December 2019 in Wuhan, China. It has caused a large global outbreak and become a significant public health issue (Lai et al., 2020).

The most recently circulating variant of SARS-CoV-2, Omicron, was first identified from an immunocompromised patient in South Africa (Machkovech et al., 2024; Wang and Cheng, 2022). Only 48 h after its detection, on 26 November 2021, the World Health Organization (WHO) officially disclosed the emergence of a novel SARS-CoV-2 variant of concern (VOC) defined as Omicron (Pango lineage B.1.1.529 and Next strain clade 21K) (Rana et al., 2022). Since then, it has become the dominant SARS-CoV-2 variant causing coronavirus disease 2019 (COVID-19). The Omicron variant has exhibited increased infectivity (Contini et al., 2023), a substantially greater number of mutations than previous VOC, and immune evasion ability, which have raised global concerns. These changes have increased the risk of reinfection and breakthrough infections with the Omicron variant (Ao et al., 2023).

Adaptive immune responses are crucial to clear SARS-CoV-2 infections and support vaccine efficacy. The adaptive immune system comprises three fundamental cell types: B cells (the source of antibodies), CD4^+^, and CD8^+^ T cells. These components play essential roles in protecting against viral infections (Primorac et al., 2022). The humoral immune response is an important arm of adaptive immunity, producing diverse antibodies against multiple epitopes that emerge with different kinetics and have diverse roles in immune protection (Carrillo et al., 2021). CD8 (cytotoxic, lethal) T cells are activated upon recognition of viral peptides presented by HLA-I class molecules following SARS-CoV-2 infection and play a critical role in reducing susceptibility to severe COVID-19, hospitalization, and death (Kombe Kombe et al., 2022). Similarly, CD4 T lymphocyte receptors bind to complexes formed by viral peptides and HLA-II class molecules, activating CD4^+^ T cells that carry out multiple functions, ranging from activation of the innate immune system cells, stimulating B cells to produce antibodies and enhancing CD8 T-cell responses (Swain et al., 2012).

A mounting body of evidence points to a critical role for SARS-CoV-2-specific T cell responses in COVID-19 recovery and modulation of disease severity. Robust T-cell responses, both quantitatively and qualitatively, are associated with milder outcomes in individuals with previous SARS-CoV-2 immunity (Heide et al., 2021). Additionally, CD8^+^ T cells are crucial in mitigating COVID-19 severity and inducing long-term immune protection. Convalescent patients who experienced mild or moderate disease exhibit notable clonal expansion of CD8^+^ T cells in bronchoalveolar lavage fluid, robust CD8^+^ T cell reactivity to SARS-CoV-2 epitopes, and rapid CD8^+^ T cell-mediated viral clearance (Peng et al., 2020; Liao et al., 2020).

Interestingly, less severe manifestations of COVID-19 are associated with coordinated antibody, CD4^+^, and CD8^+^ T-cell responses (Koblischke et al., 2020). In contrast, severe cases correlate with a lack of coordination between cellular and humoral responses, leading to delayed adaptive responses (Huang et al., 2020). The dominance of the Omicron variant and its rapid evolution into various sub-lineages have raised concerns regarding the effects of the immunity elicited by previous natural infection or vaccination, in the human population worldwide. This rapid evolution has caused the epitope variation in different Omicron sub-lineages.

To address this issue, we compared the amino acid (aa) sequences of B cell, CD4^+^, and CD8^+^ T cell epitopes in the original SARS-CoV-2 strain (Wuhan-Hu-1) with some Omicron sub-lineages recently circulated in Iran, which were subjected to whole genome sequencing in National Influenza Centre (NIC).

## Materials and methods

### Study design and sample collection

From October 2023 to August 2024, 100 SARS-CoV-2 positive oropharyngeal swab specimens (OPS) were selected from samples sent to Iran NIC for routine surveillance of influenza-like illnesses and targeted surveillance of COVID-19. At first, these specimens were subjected to a real-time reverse transcription-polymerase chain reaction (rRT-PCR) assay to reconfirm the SARS-CoV-2 positivity and to check the Ct value. Specimens with a Ct value of <30 were selected to have a proper amount of SARS-CoV-2 genome for the library preparation. Then, next generation sequencing (NGS) was performed with 2 methods, and 49 samples with high coverage (more than 70%) were selected to perform a precise phylogenetic and epitope evaluation.

### RNA extraction and rRT-PCR assay

Viral RNA was extracted from all samples using High Pure Viral Nucleic Acid kit (Roche, Germany) according to the manufacturer’s instructions. Sixty microliters of purified RNA was kept at −80°C until further use. The molecular detection of the SARS-CoV-2 infection was confirmed and the Ct value was determined by rRT-PCR test with primers and probes for E and RdRp genes (Corman et al., 2020). The positive samples with Ct value <30 were selected for NGS performance.

### Library preparation and next generation sequencing

In this study, quality control measures were implemented for all stages of the NGS procedures. Before library preparation in sample selection, and evaluation of extracted RNA. During library preparation steps including, fragmentation, adapters ligation, and amplifications. Finally, in pooling libraries and the final NGS library.

### Oxford Nanopore Technology

The DNA library was prepared using PCR tiling of targeted genomes with Midnight RT- PCR Expansion and rapid barcoding kits (EXPMRT001 and SQK-RBK110.96) (ONT, London, United Kingdom) according to the manufacturer’s instructions. After barcoding, each library was pooled with the other libraries. The pooled library was purified on a magnetic rack and cleaned using AMPure XP beads. Subsequently, an appropriate library concentration was loaded onto Oxford Nanopore MinION SpotON Flow Cells FLO-MIN106D, R9.4.1 (v9). The library was sequenced using the Oxford Nanopore MinION Mk1C device for 14 h. This duration of sequencing ensures that a comprehensive quantity of genetic material was sequenced, providing crucial high coverage needed for accurately identifying rare variants even with low-frequency mutations. During the sequencing procedure MinKNOW software controlled the Nanopore sequencing device, and performed multiple core tasks, comprising of data acquisition, analysis in real-time, basecalling, and data streaming (Kalendar et al., 2023).

### Illumina platform

After viral RNA extraction, cDNA was synthesized by Thermo Scientific Maxima H (Minus kit). The library was constructed using Nextera DNA Flex kit (Illumina, United States), according to the manufacturer’s instructions. DNA segmentation was performed using bead linked transposomes (BLT) and afterward, adapter sequences were added. Then, tagmented DNA was amplified, and the clean-up steps were done. Subsequently, the library constructed underwent probe hybridization using a Respiratory Virus Oligo Panel kit (Illumina, United States). This step was followed by probe capture, enrichment, amplification, and clean-up. After quality control assessment for library concentration by Qubit (Thermo Fisher, United States) and gel electrophoresis, the pooled library was loaded onto the Illumina Next Seq 550 machine for sequencing (Yavarian et al., 2022).

### Analysis of Nanopore and Illumina sequencing data

The quality evaluation and trimming of the raw reads were performed using Fastp. Then FASTQ files were analyzed to generate FASTA files by Medaka. Finally, SAMtools v1.20 was employed to calculate the coverage of reads and the depth at each position.

After evaluating the sequence of each 100 samples on the Nextclade-Nextstrain site,^1^ alignments were carefully examined to filter out any poor-quality sequences. Only sequences that generated >70% genome coverage (49 samples) were used for further analyses. The average sequencing depth of these reads was >60%.

To compares the sequences of these 49 strains with the origin strain (Wuhan) and viruses from the other countries, multiple-sequence alignment was performed using the BioEdit version (Version 7.2.5). The nucleotide sequence of each studied sample was submitted to the GISAID database (Global initiative on sharing all influenza data) and the accession numbers were collected.

### Phylogenetic analysis

The generated Fas file was further evaluated by Molecular Evolutionary Genetics Analysis Version 11 (MEGA11) software, and a phylogenetic tree was constructed by the neighbor-joining method using the Kimura 2-parameter model with 1,000 bootstrap values.

### The amino acid evaluation of T-cell and B-cell epitopes

The nucleotide alignment was converted to aa alignment using BioEdit software and the residue changes on B cell, CD4, and CD8 T cell epitopes were assessed in comparison to the Wuhan sequence.

Altogether, 454 major histocompatibility complex (MHC) class I-restricted CD8^+^ T cell epitopes and 280 MHC class II-restricted CD4^+^ T cell epitopes, which were identified and reported in a study using activation-induced marker (AIM) assay (Tarke et al., 2021), were evaluated for all 49 strains. Selection of continuous 42 known B cell epitopes was performed based on the study published in Scientific Reports, in which the “BepiPred-2.0” was used to identify these epitopes (Polyiam et al., 2021). The rate of fully conserved B cell, CD4, and CD8 epitopes was calculated for each strain.

## Results

### Characterization of SARS-CoV-2 isolates from Iran

In our study, 100 positive SARS-CoV-2 samples with a Ct value ≤30 using rRT-PCR were selected. These samples were subjected to NGS using the Oxford nanopore and Illumina platforms. Forty-nine sequences of SARS-CoV-2 strains that generated more than 70% genome coverage were used for further analyses and all sequences were submitted to the GISAID.

The variants were evaluated through both Nextcalde-Nextstrain and GISAID websites. The results showed 31 distinct Pango lineages. Forty-eight strains belonged to different sub-lineages of Omicron, while one strain was recombinant (Table 1).

**Table 1:** A comprehensive analysis of the 49 SARS-CoV-2 full genome sequences identified in 31 distinct lineages.

Next strain clade	Pango lineage	Frequency *N*^a^ (%)
21L	BA.2.10	1 (1.96%)
22F	GW.5	1 (1.96%)
	GW.5.3	1 (1.96%)
	FY.8	1 (1.96%)
	XBB.1.42.1	3 (5.88%)
	XBB.1.41.1	1 (1.96%)
23B	XBB.1.16	2 (3.92%)
	XBB.1.16.1	1 (1.96%)
	XBB.1.16.2	1 (1.96%)
	FU.5	3 (5.88%)
23D	FL.1.5.1	1 (1.96%)
	FL.1.5.1.1	1 (1.96%)
	FL.25	1 (1.96%)
	FL.36	6 (11.76%)
	EG.1	1 (1.96%)
	EG.4	1 (1.96%)
23H	HK.3.5	1 (1.96%)
24A	JN.1	5 (9.8%)
	JN.1.1	1 (1.96%)
	JN.1.4	1 (1.96%)
	JN.1.7	1 (1.96%)
	JN.1.13.1	1 (1.96%)
	JN.1.18	1 (1.96%)
	JN.1.22	1 (1.96%)
	JN.1.33	5 (9.8%)
24B	KP.4.2	1 (1.96%)
	KP.4	1 (1.96%)
	KP.4.1	1 (1.96%)
24C	KP.3.1	1 (1.96%)
	KP.3.3	1 (1.96%)
Recombinant	XDK.1	1 (1.96%)
Total	49 (100%)	

a *N, number.*

**Figure 1:**
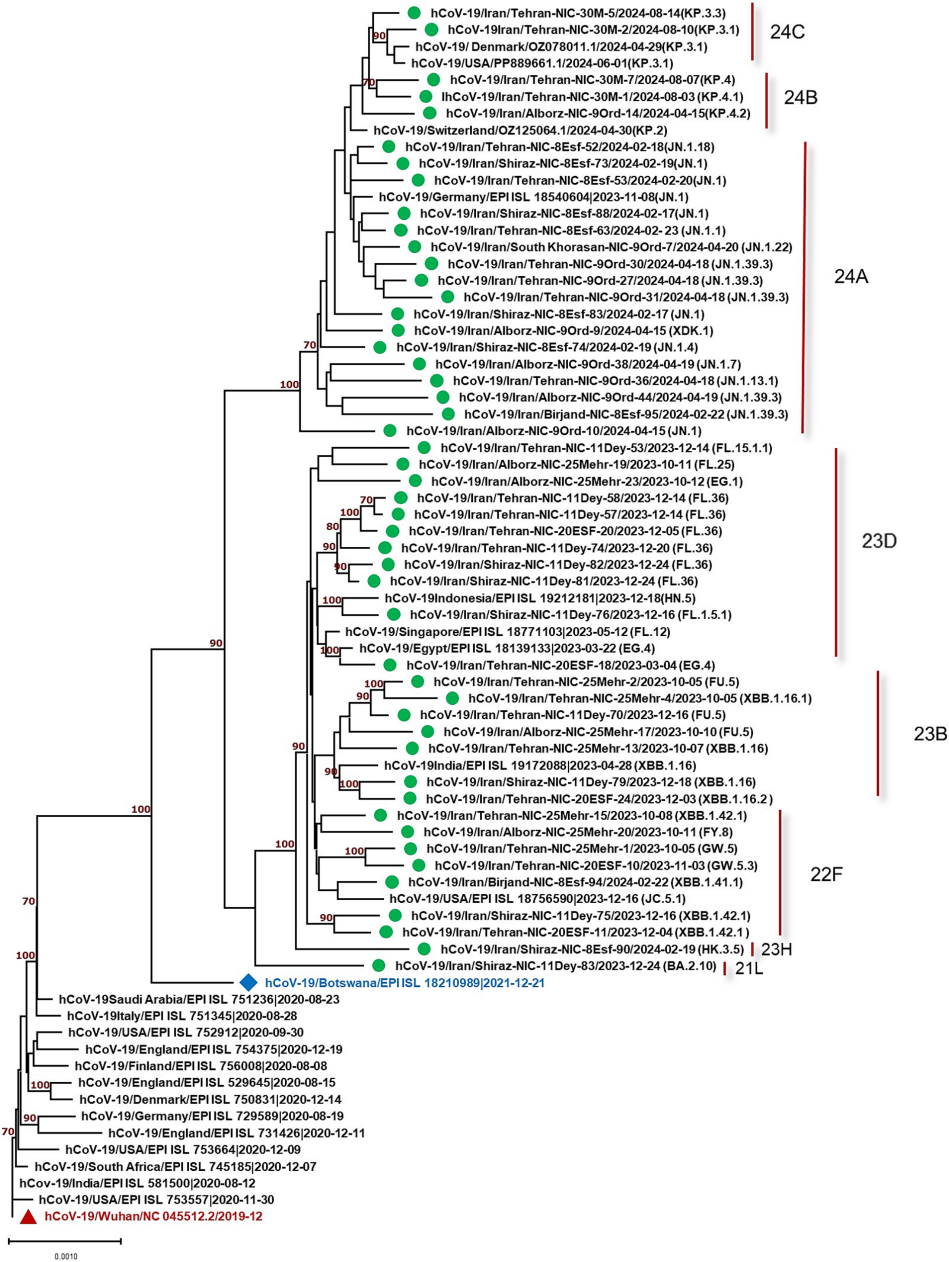
Phylogenetic analysis of SARS-CoV-2 strains circulated in Iran (showed by green circles) compared to the strains from other countries. Phylogenetic tree was constructed by the neighbor-joining method using the Kimura 2-parameter model with 1,000 bootstrap values.

The constructed phylogenetic tree (Figure 1) confirmed the variant and sub-lineages that Nextcalde-Nextstrain and GISAID websites suggested. This analysis was performed based on the whole genome nucleotide sequences of circulated SARS-CoV-2 strains in Iran, other countries, and the Wuhan strain as the root. As seen on the tree, Iranian strains fell into the same branches, with similar sub-lineages of Omicron from other countries. The phylogenetic tree exhibited 8 Nextstrain clades (21L, 22F, 23B, 23H, 23D, 24A, 24B, 24C) in 48 studied strains, and one strain (XDK.1) was recombinant. Seven viruses were clustered in clade 22F with a reference from the USA (EPI ISL 18756590). Seven strains fell into the clade 23B with a reference from India (EPI ISL 19172088). The clade 23D comprises of 11 Iranian strains which were clustered with references from Egypt (EPI ISL 18139133), Singapore (EPI ISL 18771103), and Indonesia (EPI ISL 19212181). Clade 24A was found in 16 strains (32%) that clustered with a reference from Germany (EPI ISL 18540604). Clade 24B comprises of 3 viruses that clustered with references from Switzerland (OZ125064.1). Two strains were clustered in clade 24C with references from Denmark and the USA (OZ078011.1), (PP889661.1), respectively. Clade 21L and 23H each also had one strain.

### T-cell and B-cell epitope analysis in the Omicron sub-lineages

The result of 280 CD4^+^ T cell epitopes evaluation in all 49 SARS-CoV-2 studied strains revealed that in total 65% (182/280) of these epitopes were completely conserved, in which 48% (44/92) of the spike protein epitopes and 73% (138/188) of non-spike proteins were fully conserved.

**Figure 2:**
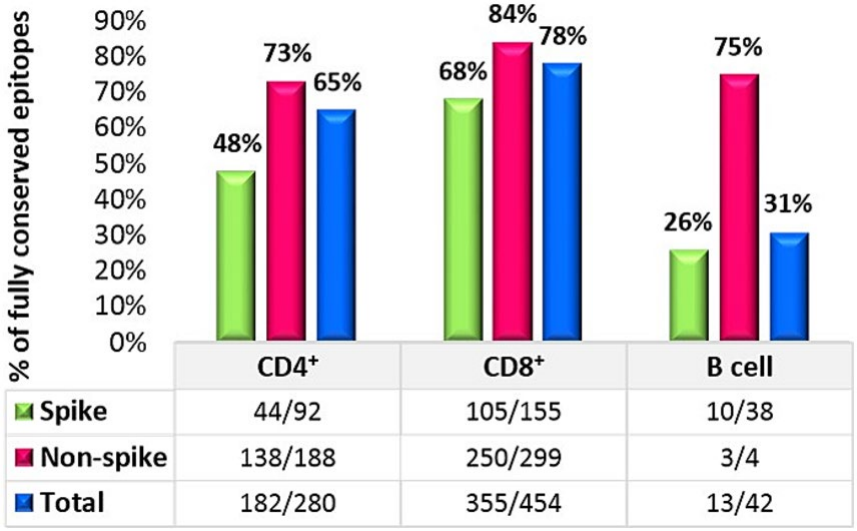
T-cell and B-cell analysis against the Omicron variant.

A similar assessment was performed for CD8^+^ T cell epitopes. Among the 454 known MHC class I-restricted CD8^+^ T cell epitopes, 78% (355/454) of all CD8 epitopes were fully conserved. The conservation rate for spike protein epitopes was 68% (105/155), while 84% (250/299) of all epitopes on non-spike proteins were conserved. The details of the non-spike CD8^+^ and CD4^+^ T cell epitope changes are listed in Table 2. Finally, an evaluation of continuous known B cell epitopes revealed that only 13 out of 42 (31%) were fully conserved, with 26% (10/38) on the spike protein, 100% (2/2) on the E protein, and 50% (1/2) on the M protein (Figure 2).

**Table 2:** The summary of T and B cell epitopes changes in non-spike proteins.

Protein name	Frequency of conserved CD4 T cell epitopes *N*^a^ (%)	Frequency of conserved CD8 T cell epitopes *N*^a^ (%)	Frequency of conserved B cell epitopes *N*^a^ (%)
nsp 3	23/27 (85%)	107/126 (85)	—
nsp 4	11/16 (69%)	29/29 (100)	—
nsp 12	13/16 (81%)	36/39 (92)	—
nsp 13	5/5 (100)	0	—
nsp 16	3/3 (100)	8/8 (100)	—
ORF 3a	10/20 (50)	15/22 (68)	—
ORF 8	20/20 (100)	—	—
E	0	0	2/2 (100)
M	25/39 (64)	27/36 (75)	1/2 (50)
N	28/42 (66)	28/39 (72)	—
Total	138/188 (73)	250/299 (83)	3/4 (75)

a *N, number.*

### T cell epitopes of spike glycoprotein demonstrate partial conservation across the Omicron sub-lineages

We evaluated the extent of conservation of T cell epitopes in the spike glycoproteins of the Omicron sub-lineages to gauge the influence of SARS-CoV-2 evolution on T cell immunity. Among the 92 CD4 epitopes found in the wild-type strain spike glycoprotein, 48 (52%) epitopes included an alteration in at least one analyzed sub-lineage. Then we evaluated 155 CD8 epitopes of which, 50 (32%) epitopes included a position reported to be mutated in at least one analyzed sub-lineage. Nevertheless, about 70% of CD4^+^ and 82% of CD8^+^ T-cell epitopes of the wild-type spike glycoprotein were conserved among different clades (Table 3). The degree of CD8^+^ and CD4^+^ T cell epitope conservation showed a slight decrease in clade 24A (74% for CD8 and 59% for CD4) and in clade 24C (59% for CD4) (Table 3). Altogether, these data suggested that T cell epitopes in the Omicron sub-lineage were considerably preserved.

**Table 3:** Relative frequency of conserved CD4 T cell, CD8 T cell, and B-cell epitopes in the spike glycoproteins of the SARS-CoV-2 variants compared to the wild-type.

Next strain clade	Pango lineage	Frequency of conserved CD4 T cell epitopes *N*^a^ (%)	Frequency of conserved CD8 T cell epitopes *N*^a^ (%)	Frequency of conserved B cell epitopes *N*^a^ (%)
21L	BA.2.10	89%	96%	65%
22F	GW.5	71%	83%	52%
	GW.5.3			
	FY.8			
	XBB.1.42.1			
	XBB.1.41.1			
23B	XBB.1.16	65%	83%	50%
	XBB.1.16.1			
	XBB.1.16.2			
	FU.5			
23D	FL.1.5.1	65%	78%	47%
	FL.1.5.1.1			
	FL.25			
	FL.36			
	EG.1			
	EG.4			
23H	HK.3.5	80%	87%	60%
24A	JN.1	59%	74%	36%
	JN.1.1			
	JN.1.4			
	JN.1.7			
	JN.1.13.1			
	JN.1.18			
	JN.1.22			
	JN.1.33			
24B	KP.4.2	70%	78%	55%
	KP.4			
	KP.4.1			
24C	KP.3.1	59%	78%	47%
	KP.3.3.3			
Recombinant	XDK.1	69%	81%	55%

a *N, number.*

### The neutralizing B cell epitopes of the spike glycoprotein are gradually disappearing within the Omicron sub-lineages

To assess the degree of B cell epitope conservation across Omicron sub-lineages, we analyzed 38 neutralizing B cell epitopes within the spike glycoprotein, consisting of experimentally confirmed epitopes from the Polyiam et al. (2021) study. Of these 38 epitopes, 28 (74%) included a position altered in at least one of the analyzed clades. Although 52% of B cell epitopes of the wild-type spike glycoprotein were conserved among different clades (Table 3). The B cell epitope conservation level was marginally lower in clade 24A (36%) (Table 3). We found that B cell epitopes were partially conserved in Omicron sub-lineages.

## Discussion

The emergence of SARS-CoV-2 in the human population introduced the COVID-19 pandemic in 2020. Since then, genome changes due to the nature of the virus, adaptation to the host, and immune response pressure, have generated different SARS-CoV-2 variants, lineages, and sub-lineages designated by various naming conventions.

This study evaluated 49 strains circulated in Iran from October 2023 to August 2024. The results showed one recombinant strain (XDK-1) and 8 clades, designated as 21L, 22F, 23B, 23H, 23D, 24A, 24B, and 24C in the studied strains. Amongst, 24A was the most predominant clade found in 16 strains (32.65%) consisting of, eight different Pango lineages (Omicron sub-lineages). The most predominant Pango lineage in this clade was JN.1. The detection of JN.1, for the first time, was reported by the USA in September 2023. The mean nucleotide differences of our JN.1 strains compared to the Wuhan strain was 0.9% which was similar to 0.5% observed in one strain (EPI ISL 18540604) from Germany. The second dominant clade was 23D with 11 studied strains including FL and EG sub-lineages with the mean diversity of 0.6 and 0.5% with the Wuhan virus. This level of alteration was similar to that of strain (EPI ISL 18139133) from Egypt, which exhibited 0.4% diversity. The variation rate of the other studied clades was also similar to the strains from other countries (Figure 1).

These findings were parallel with the other studies that showed that SARS-CoV-2 strains of similar clades and sub-lineages had the same mutations and substitutions compared to the Wuhan strain, despite circulating in different countries. Some of these mutations and substitutions have been detected in viral epitopes. Immune responses induced by SARS-CoV-2 infection and/or vaccination are comprised of antibodies, effector T cells, and memory T cell responses that target viral epitopes (Harne et al., 2023). The pressure of these immune responses may cause and/or select some epitope changes, while some epitopes stay conserved. The dynamic changes of T and B cell epitopes in the Omicron sub-lineages are unclear. To address this issue, the presented study evaluated the previously identified viral epitopes in Omicron sub-lineages in Iran. Herein, the epitopes conservation rate, based on the amino acid sequences, in spike and non-spike proteins in studied strains were compared to the Wuhan. It should be noted that, this study had some limitations. No information was available, regarding patients’ underlying diseases, immune system status, and vaccination history.

The S protein plays a vital role in the virus infection cycle and host immune responses (Rotondo et al., 2021). Our results showed that approximately 48% (44/92) of CD4 epitopes from the spike protein were fully conserved in the Omicron sub-lineages. Choi’s et al. (2022) study reported an even higher conservation rate, with 80.4% (74/92) of CD4 epitopes from the spike protein fully conserved in Omicron, but that study was conducted in January 2022 and it seems that epitopes have undergone more changes over time.

Park et al. (2022), in a study published in August 2022, examined the fractions of completely conserved T cell epitopes to provide insights into T cell responses against BA.1, BA.2, BA.2.9, BA.2.12.1, BA.4, BA.5. Their findings illustrated that the median CD4 epitopes conserved fractions were ≥73.9% for the spike epitopes. Li et al. (2022) demonstrated that most CD4 T cell epitopes were fully conserved in Omicron sub-lineages (BA.1, BA.2, BA.3, and GKA). Specifically, within BA.1, 65% (60/92) of spike epitopes were conserved, while BA.2 sub-lineage had 71% (65/92) conservation. BA.3 showed 73% (67/92) conservation, and GKA preserved 70% (64/92) of spike protein epitopes. Our study found a 70% conservation rate for these epitopes across various clades. Muik’s study reported a similar ratio (70%) for CD4^+^ cell epitopes.

For CD8 epitopes, based on our results, 68% (105/155) of spike epitopes were fully conserved. Choi et al. (2022) found that 88.4% (137/155) of these epitopes in the spike region were conserved in the Omicron sub-lineage. Park’s et al. (2022) research showed that the median conserved fractions were ≥85.3% for the spike protein. According to the study by Li et al. (2022), the conservation rates of CD8^+^ T cell epitopes in the BA.1, BA.2, BA.3, and GKA variants were 93% (114/122), 88% (107/122), 91% (111/122), and 96% (117/122), respectively. Our results demonstrated that the conservation rate of these epitopes among various clades was 82%. Muik’s et al. (2023) study reported an 80% conservation ratio for CD8^+^ cell epitopes. Our study showed that the T cell epitope conservation rate of spike protein has decreased, which suggested that the mutation rates in SARS-CoV-2, have increased as time progresses. The presented study showed that the conservation rate of epitopes was lower for clade 24A than the others. It was expected that this rate would be lower for the recent clads (clades 24B and 24C), compared to the clade 24A. This could be attributed to the time duration of 24A circulation in Iran and worldwide.

The assessment of 38 neutralizing B cell epitopes within the spike glycoprotein in different sub-lineages of the Omicron showed a conservation rate of 26% (10/38). The rest of the epitopes had changes in at least one of the analyzed strains.

The conservation rate of these epitopes among various clades was 51% (Table 3). In clade 24A, the conservation of B cell epitopes dropped slightly to 36%. Our study indicated that B cell epitopes exhibited partial conservation in Omicron sub-lineages. Different from our study, Muik et al. (2023) in a study published in August 2023, analyzed 454 unique neutralizing B-cell epitopes within the spike glycoprotein. Of these 454 epitopes, 412 (91%) included one position altered in at least one of the analyzed Omicron sub-lineages, and only 9% were fully conserved. However, the conservation rate of these epitopes among various clades was 19%. These results explained the gradual loss of cross-neutralization antibodies in the Omicron sub-lineages.

In contrast to the epitopes from the spike protein, in this study, non-spike CD4 and CD8 epitopes showed higher conservation rates, 73% (138/188) and 84% (250/299), respectively. A study performed by Li et al. (2022) showed, 82% (155/188), 86% (162/188), 88% (165/188), and 93% (174/168) of CD4 epitopes were conserved in the BA.1, BA.2, BA.3, and GKA variants, respectively. This result also showed that 92% (274/299), 96% (286/299), 93% (279/299), and 96% (286/299) of CD8 epitopes from non-spike proteins were conserved in the BA.1, BA.2, BA.3, and GKA variants, respectively.

Choi et al. (2022) demonstrated that 98.3% (294/299) of epitopes from non-spike proteins were fully conserved in the Omicron variant. Park et al. (2022) demonstrated that ≥91.9% and ≥97.0% of CD4 and CD8 epitopes from non-spike proteins were conserved in Omicron sub-lineages, respectively.

Several mutations within immunodominant N protein CD8^+^ T cell epitopes that result in complete loss of recognition have arisen independently in multiple SARS-CoV-2 lineages. Among these N protein P13L, is present in Omicron within a B*27:05 restricted CD8^+^ epitope (Carabelli et al., 2023). Given the hypothesis that VOC emerges in chronic infections, it is tempting to speculate that the presence of P13L in the Omicron reflects selection due to T cell pressure during chronic infections, in addition to the constellation of spike mutations that are likely driven by antibody pressure. We found this mutation in 13 strains in clades 22F, 23B, 23D, 24A, 24B, and 24C.

Non-spike protein B cell epitope showed a 75% (3/4) conservation rate, but we did not find a similar study that investigated these epitopes. As mentioned, the Omicron sub-lineages of SARS-CoV-2 with a B cell epitope conservation rate of 31% in our study, exhibited a significant ability to evade neutralizing antibody responses, as indicated by the research of Garcia-Beltran et al. (2022) and Hoffmann et al. (2022). This evasion is likely due to the striking enrichment of mutations at key sites in the receptor binding domain (RBD) of the spike, which are critical for neutralization by antibodies (Bhattacharya et al., 2023).

In contrast to the neutralizing antibodies that mainly recognize the surface of the spike, T cell epitopes are numerous and located across the entire spike protein of SARS-CoV-2. This suggests that most T cell responses against the spike protein target conserved epitopes, potentially restricting viral evasion from T cells (Grifoni et al., 2021; Keeton et al., 2022). Consequently, Omicron can hardly evade from T cell responses due to the frequent T cell epitopes distributed throughout both structural and nonstructural proteins. These findings are in concert with our results that showed 65 and 78% conservation rates for CD4 and CD8 epitopes, respectively.

Our findings illustrated that T cell epitopes of the SARS-CoV-2 are considerably preserved across major Omicron sub-lineages. Conservation levels varied based on epitope class (higher for CD8 vs. CD4), protein (higher for non-spike vs. spike), and clades (higher for 21L, 22F, 23B, 23H, 23D, and 24B vs. 24A and 24C). The greater conservation of CD8 epitopes than CD4 epitopes is likely due to the shorter peptide length of CD8 epitopes. Here the rate of conservation in CD4 and CD8 epitopes of spike protein were less than the non-spike proteins. This finding is consistent with the high mutation rates in the spike protein not only across Omicron sub-lineages but also from the beginning of SARS-CoV-2 evaluation.

In conclusion, the high rate of conservation in T cell epitopes of non-spike proteins among different sub-lineages of the SARS-CoV-2 Omicron variant in this study highlighted the importance of cell-mediated immunity and suggested that non-spike proteins might be more attractive targets for the second-generation SARS-CoV-2 vaccines, to offer protection against new strains with escape mutations in B cell epitopes and to prevent severe illness.

## Data Availability

The dataset presented in the study were deposited in the GISAID database with accession numbers: EPIISL19508827 to EPIISL19508874 and EPIISL19511365. All data has been released and is available to the public. Further inquiries can be directed to the corresponding authors.

## Footnotes

^1^ https://clades.nextstrain.org
